# Effective Educational Programs for Disaster Preparedness and Management: A Rapid Evidence Assessment

**DOI:** 10.1111/inr.70193

**Published:** 2026-07-09

**Authors:** Lara Delbene, Sara Mezini, Camilla Cacciabue, Roger Watson, Giuseppe Aleo, Marco Di Nitto, Milko Zanini, Gianluca Catania, Loredana Sasso, Annamaria Bagnasco

**Affiliations:** ^1^ Department of Health Sciences University of Genoa Genoa Italy; ^2^ Academic Dean Southwest Medical University Luzhou China; ^3^ Faculty of Nursing & Midwifery Royal College of Surgeons in Ireland Dublin Ireland

**Keywords:** Educational program, disaster nurse, disaster preparedness, nursing competence, nursing education, simulation training, Rapid Evidence Assessment

## Abstract

**Aim:**

To evaluate the effectiveness of educational programs on disaster preparedness and management for emergency nurses and nursing students, focusing on teaching methods that enhance readiness and competence.

**Background:**

The increasing frequency and severity of disasters require healthcare systems to be adequately prepared. Nurses, often on the frontline, must receive structured and effective training to respond to critical events with competence and resilience.

**Methods:**

A Rapid Evidence Assessment was conducted to explore and synthesize the available literature on educational interventions in disaster preparedness. Studies were identified through structured searches in international databases and were selected and evaluated using the PRISMA 2020 checklist. The quality of included studies was appraised using the CASP tool.

**Results:**

Thirty‐six studies met the inclusion criteria. Educational programs incorporating realistic simulations, virtual technologies, and interprofessional exercises were most effective in improving technical skills, confidence, and crisis management. Programs varied in format and intensity but shared common elements such as experiential learning and integration of psychological preparedness.

**Discussion:**

Educational interventions must balance theory with practice and adapt to diverse cultural and contextual needs. Simulations and technology‐enhanced learning emerge as critical strategies to reinforce emergency competencies.

**Conclusion:**

Educational programs in disaster preparedness positively influence nurses’ readiness, skills, and confidence. Integrating these interventions into nursing education is essential to prepare a responsive and resilient workforce.

**Implications for Nursing:**

Ongoing and structured education strengthens nurses’ ability to respond effectively during disasters, promoting professional growth and emotional resilience.

**Implications for Health Policy:**

Policies should support the mandatory inclusion of disaster preparedness training in nursing curricula and continuing education. National frameworks must prioritize standardized educational models to ensure healthcare system resilience and nurse preparedness worldwide.

## 1 Background

In recent decades, the cyclical occurrence of natural and man‐made disasters has endangered human lives and material assets worldwide (Magi et al. 2023). This phenomenon is attributed to factors such as rapid population growth, overcrowding, intensive urbanization, climate change, the increasing threat of terrorism, and armed conflicts. According to the Emergency Events Database, in 2024, a total of 393 natural hazard–related disasters were recorded worldwide, resulting in 16,753 deaths, affecting approximately 167.2 million people, and causing economic losses estimated at USD 241.95 billion (EM‐DATE 2025). Such events not only result in death and disability but also negatively impact the quality of life and the economic and social stability of the affected communities (World Health Organization 2019).

Nurses, one of the professional groups most involved in disaster management, play crucial roles across all phases of the disaster cycle, including mitigation/prevention, preparedness, acute response, recovery/rehabilitation, and longer‐term community support (Goniewicz et al. 2021; Shi et al. 2025). This responsibility requires continuous and specialized training in emergency and trauma management techniques to ensure rapid and effective responses (Goniewicz et al. 2021). For this reason, disaster preparedness and response must be an integral part of nursing competencies, necessitating standardized training at a global level (Koca and Arkan 2020).

As disaster nursing is an emerging field, nurses face various challenges, including inadequate levels of preparedness, poor training, lack of research, and ambiguity regarding their roles during disasters. Therefore, it is essential to identify the necessary competencies for specific groups and emergency contexts, as well as to promote greater nursing research to improve preparedness and effectiveness in managing global disasters (Chegini et al. 2022). In line with this, the WHO has developed an operational framework based on a global approach that includes prevention, preparedness, response, and recovery, promoting an inclusive model that respects ethical considerations (World Health Organization 2019).

Complementing the WHO operational framework, the International Council of Nurses, in collaboration with the WHO, has published the *Core Competencies in Disaster Nursing (Version 2.0)*, translating the disaster cycle into a structured, competency‐based reference articulated through progressive levels of nursing practice and operational domains including communication, incident management, safety, ethical practice, and recovery (International Council of Nurses; World Health Organization 2019).

Recent mapping reviews have further delineated disaster preparedness competency domains for emergency nurses, confirming the centrality of structured frameworks in guiding education and training (Motsepe and Schmollgruber 2025). Despite these frameworks, evidence indicates that disaster preparedness levels among nurses remain variable and often suboptimal, particularly in high‐risk regions (Labrague and Hammad 2024)

Nurses’ preparedness requires a structured approach to understand disaster‐related threats and respond promptly while managing limited resources and making critical decisions under pressure (Su et al. 2023). The psychological pressure and stress that healthcare providers face during disasters often have significant consequences on their mental health, leading to symptoms of insomnia, anxiety, and depression (Hawsawi 2022).

Continuous and well‐structured training enhances responses to critical events and ensures that healthcare personnel are prepared for future challenges, making integrated education with standardized key competencies at the national level essential to strengthen resilience and readiness in emergency situations (Littleton‐Kearney and Slepski 2008). High‐fidelity simulations and the use of technology have been shown to offer more engaging training compared to traditional methods, although it remains complex to identify which is the most effective method across different contexts, as the needs and resources of populations vary significantly (Bosak et al. 2023).

The literature evaluating the impact of educational programs on disaster preparedness among emergency nurses is limited, and the lack of access to international research, particularly in developing countries, hinders effective emergency management. To improve this situation, it is crucial for local officials and hospital administrators to provide adequate resources and for emergency nurses to be actively involved in developing local or national guidelines and emergency plans (Al‐Qbelat et al. 2022).

Although the number of educational interventions in disaster nursing has increased in recent years, the existing evidence remains fragmented across study designs, educational formats, outcome measures, and geographic contexts.

This Rapid Evidence Assessment (REA) addresses this methodological and contextual gap by providing a structured overview of educational approaches, outcome domains, and implementation contexts, thereby clarifying the current evidence landscape and identifying areas requiring further research.

## 2 Aim

The aim of this study is to critically evaluate the effectiveness and efficiency of existing educational programs on disaster preparedness and management targeted at emergency nurses and nursing students, with a specific focus on their impact in enhancing competencies for crisis response.

To achieve the primary aim, this study also pursues the following secondary objectives:

1. Analyze the extent to which educational interventions improve nurses’ readiness and ability to respond promptly and effectively in emergency situations.
2. Identify gaps in the current body of research concerning disaster preparedness programs for nursing professionals.
3. Propose a comprehensive educational framework that promotes innovative and targeted training approaches for more effective disaster preparedness in nursing practice.

## 3 Methods

This REA was reported in accordance with the PRISMA 2020 (Preferred Reporting Items for Systematic Reviews and Meta‐Analyses) guidelines (Page et al. 2021) to ensure methodological rigor and transparency in systematic review reporting. The PRISMA 2020 checklist was used throughout the development and reporting of the review. A completed version of the checklist is available as supplementary material (see Appendix A).

### 3.1 Research Strategy

The REA method was used (Varker et al. 2015). This approach has proven particularly suitable for assessing the effectiveness and efficiency of educational programs for disaster preparedness for nursing students and emergency nurses. The SCOPUS, PUBMED, and CINAHL databases were searched to conduct this REA.

The research question that guided the review was: “What are effective and efficient educational programs for disaster preparedness and management among emergency nurses and nursing students?”

Based on the research question, the population, exposure, and outcome (PEO) framework was used to identify relevant keywords for the development of the search strategy. These keywords were combined using the AND and OR Boolean operators to construct comprehensive search strings. The components of the PEO framework and the corresponding keywords used for each database are shown in Table 1.

**TABLE 1:** PEO framework and research string.

	Description	Keywords
P (Population)	Graduate nurses working in emergency and urgent care departments, and nursing students	“emergency nurse” OR “nursing student” OR “graduate nurse” OR “urgent care nurse” OR “disaster nurs*”
E (Exposure)	Educational programs focused on disaster preparedness and management	“educational program” OR “training program” OR “workshop” OR “disaster preparedness training” OR “disaster preparedness”
O (Outcome)	Effectiveness and efficiency in disaster management and response capabilities	“effects” OR “impact” OR “consequences” OR “outcome” OR “efficiency” OR “effectiveness”

*Note*: PEO = Population, Exposure, Outcome. The terms were combined using Boolean operators (AND/OR) to construct the search strings.

### 3.2 Inclusion and Exclusion Criteria

To ensure that the included studies were relevant and of high quality, rigorous inclusion and exclusion criteria were applied. We selected quantitative studies that assessed the effectiveness of nursing training programs in disaster management. The participant sample included graduate nurses working in emergency departments and nursing students, and the time frame considered encompassed studies published between January 2000 and April 2024. This time range allowed for the inclusion of both recent studies and those conducted in the context of relevant catastrophic events, thereby offering a comprehensive perspective on the development of disaster preparedness competencies.

Qualitative studies and those involving only non‐nursing professionals were excluded from the review. Direct contact with authors for clarifications or further information was not planned, in line with the limitations of the REA method (Varker et al. 2015).

### 3.3 Study Selection Process

The study selection process was conducted rigorously by two reviewers using the Rayyan website (Ouzzani et al. 2016). In this phase, each reviewer independently examined the titles and abstracts of all articles retrieved from the various databases. Studies that met the inclusion criteria or appeared potentially relevant subsequently underwent full‐text screening. During both the abstract and full‐text screening phases, any disagreements between the two reviewers regarding the eligibility of specific studies were initially addressed through direct discussion. If consensus could not be reached, a third independent reviewer was consulted to make the final decision. This multistep review process ensured an objective and rigorous assessment of the studies, minimized selection bias, and upheld a high level of methodological transparency and reliability throughout the selection process.

### 3.4 Quality Assessment

Each included study was independently appraised by two reviewers using the appropriate CASP checklist according to study design. For each domain, a judgment (Yes/No/Can't tell) was recorded. Disagreements were resolved through discussion or consultation with a third reviewer when necessary. In line with CASP guidance, no overall numerical score was calculated.

Studies were classified as high, average, or low methodological quality using predefined decision rules based on the extent to which key methodological standards were met. Attention was paid to clarity of aims and design, appropriateness and validity of outcome measures, rigor of data collection and analysis, control of potential bias, and transparency of reporting.

The quality classification was used to inform the interpretation of findings rather than as an exclusion criterion. Overall, most studies were rated as average quality, six were classified as high quality, and one as low quality.

### 3.5 Description of Included Articles

Details about the included articles are shown in Table 2 to provide a comprehensive overview of each study. The extracted information included the study title, authors, year of publication, methodological design, study population, and primary research objective. Additionally, we reported the study setting and location, as well as a detailed description of the interventions analyzed or implemented.

**TABLE 2:** Summary of studies on educational interventions for disaster preparedness in nurses and nursing students.

Title	Author and year	Study design	Objective	Setting and country	Population	Focus/Key themes	Intervention	Results	Quality appraisal
The Effect of the Disaster Management Training Program Among Nursing Students	Koca B., Arkan G. (2020)	Randomized controlled trial (two‐group comparison: experimental vs. control group)	To assess the effectiveness of an educational program based on the Jennings Disaster Nursing Management Model and supported by a learning management system in enhancing nursing students’ perceptions of disaster preparedness and disaster response self‐efficacy (DRSES)	Educational setting, Turkey	*N* = 235 third‐year nursing students (experimental group = 127; control group = 108)	Disaster nursing education; disaster preparedness; DRSES; model‐based education; e‐learning	Training program based on the Jennings Disaster Nursing Model (pre‐disaster, during disaster, post‐disaster, and recovery), assisted by a learning management system (six modules).	After the intervention, the experimental group showed a significant increase in disaster preparedness perception and DRSES compared with the control group (*p* < 0.05). The intervention explained 33.1% of the variance in disaster preparedness perception (*R* ^2^ = 0.331) and 31.7% of the variance in DRSES (*R* ^2^ = 0.317), with a moderate effect size.	Average
Comparison of the Effect of Lecturing and Tabletop Exercise Methods on Level of Preparedness of Nurses	Mirzaei et al. (2020)	Quasi‐experimental study with pretest, posttest design (intervention vs. control group comparison)	To compare the effects of lecturing alone versus lecturing combined with a tabletop exercise on nurses’ preparedness for natural disasters	Hospital setting, Iran	*N* = 74 nurses working in hospital wards (Group A: lecturing, *n* = 37; Group B: lecturing + tabletop exercise, *n* = 37)	Disaster preparedness; nursing education; teaching methods; tabletop exercise; knowledge, attitude, and performance	Participants in Group A received an eight‐hour disaster preparedness training delivered through traditional lecturing. Participants in Group B received the same eight‐hour lecturing program supplemented with a two‐hour tabletop exercise based on an earthquake scenario. Outcome measures were collected at baseline (pre‐intervention), immediately after the intervention, and at a one‐month follow‐up.	Both methods significantly improved nurses’ knowledge, attitude, and performance (*p* < 0.001). Improvements were consistently greater in the lecturing–tabletop exercise group, with higher total preparedness scores at posttest and follow‐up.	Average
Game‐Based vs. Case‐Based Training for Increasing Knowledge and Behavioral Fluency of Nurse Students	Masoumian Hosseini et al. (2022)	Quasi‐experimental study with pretest–posttest design (intervention vs. control group comparison)	To compare the effectiveness of game‐based training (GBT) incorporating precision teaching versus case‐based training (CBT) in improving nursing students’ knowledge and behavioral fluency in crisis and disaster management	Educational setting, Iran	*N* = 60 third‐year nursing students who had completed clinical clerkship (intervention and control groups)	Disaster nursing education; behavioral fluency; knowledge retention; clinical decision‐making in disaster scenarios; game‐based learning; case‐based learning	The experimental group received a disaster‐themed GBT designed according to precision teaching principles, including frequency building, defined fluency criteria, and structured performance monitoring. The control group received CBT. The educational program consisted of a 5‐week theoretical phase followed by a 4‐week clinical internship. Outcomes were assessed using the Disaster Nurses’ Knowledge Questionnaire and an Objective Structured Clinical Examination (OSCE) conducted across five stations based on the Pazin model.	No significant baseline differences between groups. GBT significantly improved knowledge scores compared with CBT (*p* < 0.001). Behavioral fluency significantly higher in GBT at 1 week and 1 month post‐intervention (*p* < 0.001). No significant decline in GBT between 1 week and 1 month (*p* = 0.056). The CBT group showed a significant performance decline at 1 month (*p* < 0.0001).	Average
A Randomized Trial of Teaching Clinical Skills Using Virtual and Live Standardized Patients	Triola et al. (2006)	Randomized controlled trial (pre–post design with two‐group comparison)	To determine the effectiveness of virtual patient (VP) simulations compared with live standardized patient (SP) simulations in improving clinical skills, diagnostic ability, knowledge, and attitudes among health care providers	Educational setting, USA	55 health care providers (45% registered nurses, 15% physicians, 40% other health professionals); Control group (*n* = 32), Intervention group (*n* = 23)	Clinical skills training; virtual patients vs. standardized patients; disaster‐related psychosocial care; screening and diagnostic skills; simulation‐based learning; CME education	Participants were randomized to receive either 4 live standardized patient (SP) cases (control group) or 2 live SP cases plus 2 virtual patient (VP) simulations (intervention group). The workshop focused on screening, diagnosing, and treating psychosocial sequelae of disasters (PTSD, acute stress disorder, sub‐diagnostic distress, bereavement). Apart from the simulation modality, all other aspects of the course were identical. Pre‐ and post‐workshop assessments were conducted using Likert‐scale questionnaires and clinical vignettes.	Both groups showed equivalent improvements in comfort level, preparedness to respond, screen, and care for patients (all *p* > 0.05). No significant differences were found in subjective ratings of effectiveness between VP and SP modalities (*p* = 0.79). Improvements in diagnostic abilities were equivalent between groups. In one sub‐diagnostic stress case, the VP group showed greater improvement approaching statistical significance (*p* = 0.054). Overall, VP and SP modalities demonstrated comparable educational effectiveness.	Average
Competency in Chaos: Life‐Saving Performance of Care Providers Utilizing a Competency‐Based, Multiactor Emergency Preparedness Training Curriculum	Scott et al. (2013)	Educational intervention study (pre–post evaluation; no control group)	To develop and evaluate a competency‐based, multiactor Emergency Preparedness Training (EPT) curriculum and measure its impact on care providers’ knowledge, skills/comfort, and performance during a multipatient simulated disaster	Educational Setting, USA	Care providers and trainees *N* = 39 (24 medical students; 7 physicians; 7 nurses; 1 emergency manager)	Competency‐based disaster training; measurable performance objectives; multiactor high‐fidelity simulation; mass casualty/chaotic environment; team performance; triage/communication/PPE; debriefing with video feedback	One‐day (8‐hour) EPT course with didactic + small‐group exercises (team building, communication, triage/START) and a 6‐minute multiactor clinical disaster scenario (human simulators + ≥10 actor patients). Curriculum built around 9 learning objectives, 18 competencies (across 5 competency domains), and 34 performance objectives. Teams completed the scenario, received facilitator‐led video debriefing, then repeated the same scenario. Assessments included an online pretest (Likert self‐assessment + 23 MCQs), immediate posttest (same self‐assessment + cognitive MCQs + course evaluation), and follow‐up posttest at 4–6 months; scenario performance objectives were observed and recorded by trained observers with video‐assisted checklist completion.	Cognitive knowledge improved significantly from pretest mean 12.3 (*SD* 3.8) (∼51% correct) to post‐training mean 18.5 (*SD* 2.2) (∼77%), *p* < 0.01. Self‐assessed “Overall Skill” increased from 63.3/100 to 83.4/100 and “Overall Knowledge” from 49.3/100 to 78.7/100 (*p* < 0.01). In the disaster scenario, 23/34 performance objectives were completed by ≥50% of teams on first attempt; performance improved after debriefing (31/34 objectives completed by ≥50% teams on second attempt). Most teams were able to resuscitate unstable simulators and all teams prevented anthrax exposure on the second attempt (8/9 resuscitated two simulators; 9/9 prevented anthrax exposure). Course evaluation was very high (overall rating: ∼96/100 reported).	Average
Educating Nursing Students for Cultural Competence in Emergencies	Kula et al. (2021)	Randomized controlled trial (pre–post design with two‐group comparison)	To test whether an online culturally informed intervention embedded in the curriculum increases nursing students’ cultural competence in emergencies across four domains (attitudes, knowledge, skills, encounters)	Educational setting, Israel	Undergraduate nursing students; initially randomized *n* = 186, final matched analytic sample *n* = 72 (intervention *n* = 34; control *n* = 38)	Cultural competence in emergencies; emergency preparedness education; online learning; cultural competence domains; attitudes, knowledge; skills; encounters	The intervention consisted of a culturally informed online program delivered in two 60‐minute sessions and integrated into the curriculum; it included seven units covering definitions and challenges of emergencies plus cultural competence (attitudes, knowledge, and skills) and summary, using recorded expert lectures and reflective/self‐monitoring exercises and incorporating qualitative insights from key informants. The control group received an equivalent two‐session online program of similar structure focused on general clinician–patient communication and community resilience in emergencies, without cultural competence content; both groups completed pre‐ and post‐intervention assessments.	The intervention produced a significant improvement in the knowledge domain of cultural competence compared with the control group at post‐intervention (between‐group difference in post‐intervention knowledge controlling for baseline, F(1, 69) = 3.05, *p* ≈ 0.02). The effect on the skills domain approached significance (F(1, 68) = 3.33, *p* = 0.07). No significant group differences emerged for attitudes (F<1) or encounters (F(1, 69) = 1.77, *p* = 0.19). Across both groups and domains, post‐intervention cultural competence ratings were higher than pre‐intervention ratings (main time effect *p* <0.001), and attrition/matching issues reduced the final sample substantially.	High
Hospital Preparedness for Ebola Virus Disease: A Training Course in the Philippines	Carlos et al. (2015)	Pre–post training evaluation study (no control group)	To develop, deliver, and evaluate a workshop that could rapidly prepare large numbers of hospital‐based health professionals in the Philippines to detect, isolate, and safely manage Ebola virus disease (EVD), using a train‐the‐trainer team strategy	Hospital setting, Philippines	364 health professionals trained (doctors, nurses, medical technologists, and others)	Ebola preparedness; infection prevention and control; screening and triage; PPE donning/doffing; hospital isolation and patient flow; laboratory biosafety; waste management; staff safety; train‐the‐trainer/team approach; rapid scale‐up training in resource‐limited settings	A 3‐day workshop collaboratively developed by DOH and WHO, consisting of 18 lectures and 10 practical/small‐group sessions, including repeated PPE donning/doffing practice (with contamination simulation using red paint), screening/triage case exercises, isolation unit planning, transport, waste management, and a separate specialized stream for medical technologists (lab biosafety, specimen handling, venepuncture with PPE).	Knowledge increased significantly (*p* < 0.009) from median 7/10 (IQR 6–8) pre‐workshop to median 9/10 (IQR 8–9) post‐workshop. The proportion answering all 10 questions correctly increased from 2.8% to 22.5%. Confidence in safely caring for an EVD patient increased significantly (*p* = 0.018); post‐training 87.2% agreed/strongly agreed they could be safe caring for an EVD patient, and 96.4% reported feeling more prepared to screen/manage EVD. Knowledge regarding transmission improved but remained suboptimal (post 59.1% correct for the transmission routes item). Workshop evaluations were high (overall rated excellent/good by the majority).	Average
The Influence of Saudi National COVID‐19 Preparedness Programs on Triage Decision‐Making Skills of Healthcare Practitioners During the 2020 Peak of the 1st Wave of COVID‐19	Alzahrani and Al‐Moteri (2022)	Cross‐sectional correlational study (one‐group posttest‐only design)	To identify the effect of hospitals’ COVID‐19 preparedness educational efforts (TTP, DPP, mock drills) on triage decision‐making skills of ER healthcare practitioners during the COVID‐19 crisis	Hospital Setting, Saudi Arabia	*N* = 213 ER healthcare practitioners (45 physicians, 168 registered nurses)	Triage decision‐making; cognitive skills; critical thinking; confidence; intuition; COVID‐19 preparedness; disaster training; ER patient flow	Participation in hospitals’ COVID‐19 preparedness educational efforts included attendance at a triage training program, consisting of a one‐day online course focused on prioritization and triage principles, a disaster preparedness program aimed at introducing healthcare professionals to national emergency systems and their roles during disasters, and involvement in mock drills, defined as practical disaster response simulations designed for emergency room first responders.	Participation in the triage training program was significantly associated with higher scores across all domains of the Triage Decision‐Making Inventory, including cognitive abilities (*p* = 0.01), critical thinking (*p* = 0.03), confidence *(p* = 0.01), and intuition (*p* = 0.02). Participation in the Disaster Preparedness Program was significantly associated only with cognitive abilities (*p* = 0.04). Involvement in mock drills was significantly associated with higher confidence scores (*p* = 0.03). Overall, critical thinking and confidence demonstrated higher mean scores compared with cognitive and intuition skills.	Average
Effect of Virtual Reality Simulation Training on the Response Capability of Public Health Emergency Reserve Nurses in China: A Quasi‐Experimental Study	Zhang et al. (2021)	Prospective quasi‐experimental study with control group	To develop a virtual reality simulation training program incorporating COVID‐19 cases and evaluate its effectiveness in improving the emergency response capability of reserve nurses facing public health emergencies	Hospital setting, China	120 reserve nurses (intervention group *n* = 60; control group (*n* = 60)	Emergency preparedness; virtual reality simulation training; disaster preparedness; pandemic response capacity; emergency care capability; COVID‐19 response training	3‐month training. The control group received conventional training, while the intervention group participated in virtual reality training combined with skills training.	The intervention group showed significantly higher post‐intervention scores in theoretical knowledge, emergency care capability, and total disaster preparedness compared with the control group (*p* < 0.001). Disaster preparedness knowledge and skills subscales significantly improved (*p* < 0.001). No significant difference was found in post‐disaster management (*p* > 0.05). Technical skills scores were slightly higher in the control group (*p* < 0.01).	Average
Simulated Patient Environment: A Training Tool for Healthcare Professionals in COVID‐19 Era	Babu et al. (2021)	Comparative educational evaluation study (pre–post self‐assessment)	To implement and evaluate an in‐situ simulation model using a simulated patient environment to improve preparedness and control measures for managing COVID‐19 (and other respiratory infection outbreaks), with emphasis on screening, investigation pathway, notification to public health systems, infection control, patient care, and safety	Hospital Setting, China	*N* = 50 healthcare workers (16 doctors and epidemiologists; 23 nurses; 11 other healthcare workers)	In‐situ simulation; outbreak preparedness; screening and triage workflow; infection prevention and control practices; interdisciplinary coordination; personal protective equipment practices; emergency response readiness in real clinical environment	The program consisted of an initial briefing in the form of continuing medical education on outbreak management guidelines, distribution of official forms and standard operating procedures, rehearsal and use of stage‐specific observational checklists during the simulation, administration of a pretest questionnaire to assess baseline knowledge/attitude, execution of an in‐situ simulation in the screening area covering registration, clinical assessment, nasopharyngeal swab collection, safe transport of specimens to the laboratory, infection control and biomedical waste procedures, debriefing, and a posttest plus post‐simulation participant feedback.	Knowledge/attitude scores improved significantly from pre‐ to post‐intervention for all professional groups with very high statistical significance (paired *t*‐test *p* < 0.001). Reported mean scores increased from 33.44 to 47.50 among doctors and epidemiologists, from 38.70 to 49.35 among nurses, and from 15.45 to 35.45 among other healthcare workers (all *p* < 0.001). Participant feedback was predominantly positive, with “Excellent” or “Good” ratings reported in roughly 92%–94% of responses across feedback items, indicating high perceived relevance, improved expertise, increased confidence, and adequacy of materials to follow protocol.	Average
Interprofessional Disaster Simulation During the Covid‐19 Pandemic: Adapting to Fully Online Learning	Wong et al. (2022)	Quasi‐experimental study with pretest, posttest design (intervention vs. control group comparison)	Provide an interactive and collaborative learning activity in response to a public health disaster	Educational setting, Hawaii	*N* = 63 interprofessional students (upper‐level pre‐licensure nursing students, master's/doctoral public health students, and master's social work students). Comparator: pooled in‐person cohorts *N* = 150 from three earlier face‐to‐face simulations	Emergency preparedness; interprofessional education; online simulation; disaster triage; outbreak investigation; disaster response; teamwork, communication, ethical decision‐making; feasibility of real‐time online collaboration	Disaster Aftermath Interprofessional Simulation converted to fully online format (launched April 3, 2020). Three phases: disaster triage after a tsunami, population‐focused response to aftermath, and outbreak management in an emergency shelter (including attack rates/attributable risk and epidemic curve/pathogen identification). Required pre‐work on disaster triage, emergency response, and outbreak investigation. Delivery via Zoom main room + breakout rooms (15 rooms for 63 students) and shared Google Docs/Sheets for team tasks; large‐group debrief after each phase co‐facilitated by interprofessional faculty; materials adapted into web‐based documents and beta‐tested before delivery.	Interprofessional collaborative skills increased significantly in the online cohort based on Interprofessional Collaborative Competency Attainment Survey total mean scores (retrospective pre–post). Online students’ mean overall total score was higher than in‐person both pre (online 4.12 vs. in‐person 3.73; *p* = 0.0001) and post (online 4.50 vs. in‐person 4.25; *p* = 0.0020). All simulation learning outcomes were met. Satisfaction with ability to work through the simulations was significantly higher online than in‐person (4.27 vs. 3.99; *p* = 0.0049), while two other post‐activity items showed no significant differences. Qualitative feedback themes emphasized teamwork/collaboration, communication, and triage/ethical dilemmas; a key limitation reported was unstable internet connectivity.	Average
Enhancing Nurses' Disaster Management and Preparedness: Evaluating the Effectiveness of an Online Educational Program Through a Quasi‐Experimental Study	AlOtaibi et al. (2024)	Quasi‐experimental one‐group pretest–posttest design	To determine the effectiveness of a newly developed online educational program in improving nurses’ familiarity and attitudes toward disaster management and preparedness	Hospital setting, Saudi Arabia	*N* = 88 registered nurses	Disaster preparedness; disaster management competencies; emergency preparedness knowledge; nursing attitudes; online education; healthcare resilience	A structured 10‐hour online educational program delivered over two consecutive days (5 hours/day). The program included six modules covering disaster management fundamentals, incident command systems, ethical triage issues, isolation and quarantine, decontamination, communication during disasters, psychological issues, special populations, and critical resource management. Teaching strategies included lectures, presentations, case studies, group discussions, video demonstrations, and interactive activities.	Slight improvements observed in familiarity and attitudes toward disaster preparedness; overall familiarity increased from 3.16 ± 1.39 (pre) to 3.26 ± 1.18 (post), and attitudes increased from 2.26 ± 0.34 to 2.29 ± 0.31. However, no statistically significant differences were found between pre‐ and post‐intervention assessments (*p* > 0.05). No significant demographic differences except for educational level influencing familiarity.	Average
In‐Person and Telemedicine Course Models for Disaster Preparedness: A Comparative Analysis	Dorigatti et al. (2018)	Comparative quasi‐experimental study (pre–post design; nonrandomized comparison of in‐person vs. telemedicine cohorts)	To compare student performance in face‐to‐face versus telemedicine disaster preparedness courses and validate telemedicine as an effective training modality	Continuing professional education setting, Brazil	Mixed healthcare professionals (physicians, nurses, medical students, firefighters); telemedicine cohort: 157 enrolled (44 completed pre–post assessment); in‐person cohort: 1,398 enrolled (914 completed full pre–post testing)	Disaster preparedness training; telemedicine education; videoconferencing; continuing professional education; knowledge acquisition in disaster response	Two delivery models of the Advanced Disaster Medical Response (ADMR) course: (1) In‐person 8‐hour course delivered in one day; (2) Telemedicine model consisting of nine 1‐hour weekly videoconference sessions over nine weeks. Both used identical curricular content. Knowledge assessed via 10‐item multiple‐choice pre‐ and posttest questionnaire (score range 0–10).	Both modalities demonstrated significant knowledge increase from pre‐ to posttest (*p* < 0.001). Telemedicine: mean increased from 6.95 ± 1.60 to 8.20 ± 1.34. In‐person: mean increased from 6.51 ± 1.68 to 8.69 ± 1.22. No statistically significant difference in posttest scores between modalities (*p* = 1.0). In‐person course showed greater gain between pre–post scores (*p* < 0.05). High satisfaction reported in telemedicine group.	Average
Effect of Operational Exercises on Nurses' Competence in Dealing with Disaster	Aliakbari et al. (2022)	Quasi‐experimental study with pretest, posttest design (intervention vs. control group comparison)	To investigate the impact of implementing an operational exercise (empowerment) program on nurses’ disaster response competence	Hospital setting, Iran	*N* = 70 nurses (35 intervention; 35 control)	Disaster nursing competency; preparedness through exercises; hospital incident command system; competencies across management, ethics/legal, personal, teamwork, technical	Empowerment program for intervention group including an educational workshop, tabletop exercise (scenario‐based, hospital incident command system approach with defined roles), and an operational exercise/maneuver coordinated with the hospital crisis committee; control group received no described intervention beyond usual practice.	Baseline total competence similar between groups (control 132.57 ± 24.64 vs. intervention 121.97 ± 19.95; *p* = 0.052). Immediately post‐intervention and at 3 months, intervention group competence significantly higher than control (187.11 ± 19.72 and 187.25 ± 19.72 vs. control 128.31 ± 21.32 and 128.31 ± 21.35; both *p* < 0.001). Competence increased significantly in all domains (management, ethics, personal, teamwork, technical) in intervention group (*p* = 0.001), no significant change in control (*p* > 0.05). Group × time interaction significant (*p*<0.001).	Average
Effect of Educational Program on Knowledge, Skills, and Personal Preparedness for Disasters Among Emergency Nurses: A Quasi‐Experimental Study	Al‐Qbelat et al. (2022)	Quasi‐experimental study with one‐group pretest–posttest design	To evaluate the effect of an educational program on knowledge, skills, and personal preparedness for disasters among emergency nurses	Hospital setting, Jordan	*N* = 50 registered emergency nurses	Disaster preparedness; emergency nursing; disaster education; knowledge acquisition; triage; decontamination; trauma support; psychological management; online training	8‐hour online educational program delivered over 1 week via Zoom (two 4‐hour sessions). Five main topics: disaster preparedness concepts, START triage, decontamination and PPE in CBRN events, BLS/CPR, advanced trauma support and psychology of disaster. Teaching strategies: PowerPoint, videos, scenarios, group discussions.	Significant improvements pre–post intervention: Knowledge (*t* = 4.79, *p* ≤ 0.001), skills (*t* = 6.66, *p* ≤ 0.001), personal preparedness (*t* = 9.56, *p* ≤ 0.001), total DPET (*t* = 7.20, *p* ≤ 0.001). Scores increased but remained at moderate level post‐intervention.	Average
Psychological First Aid Training in Disaster Preparedness for Nurses Working With Emergencies and Traumas	Said et al. (2022)	Quasi‐experimental nonequivalent controlled pre–post study with two groups	To evaluate the effects of a modified psychological first aid (PFA) training program on nurses’ psychological preparedness for emergencies and disasters	Hospital setting, Palestine	*N* = 150 nurses (75 intervention; 75 control)	Psychological preparedness for disasters; PFA competencies (knowledge/skills/attitudes); self‐efficacy; self‐esteem; optimism; trait anxiety; PTSD; culturally tailored psychosocial support	Modified face‐to‐face RAPID‐PFA program (9 hours total), delivered over 5 weeks (weekly 2‐hour sessions, final 1‐hour). Methods: interactive lectures, group discussion, simulation + role‐playing using Palestinian‐relevant disaster/conflict scenarios; added emphasis on connectedness/social support/faith. Trainer: licensed psychologist (MSF). Control: wait‐listed, no training during study period.	Primary outcome (PPDTS): significant group‐by‐time effect (*p* = 0.013). Adjusted posttest means: intervention 37.81 vs. control 32.64. Secondary outcomes: significant group‐by‐time effects for optimism (*p* = 0.009), self‐esteem (*p* = 0.008), self‐efficacy (*p* = 0.033). Trait anxiety showed significant improvement (group‐by‐time *p* < 0.001). PFA evaluation subscales improved significantly (attitudes *p* = 0.003; skills *p* = 0.004; knowledge *p* < 0.001). Effect size: large (Cohen's *d* = 1.41).	High
Using Tabletop Exercises to Evaluate Nurses' Clinical Performance of Hazardous Materials Disaster Management: A Cross‐Sectional Study	Chiang et al. (2020)	Cross‐sectional study	To identify nurses’ competence in hazardous materials (Hazmat) disaster emergency response and determine factors influencing performance in tabletop exercises	Military hospital setting, Taiwan	*N* = 161 registered nurses	Hazmat disaster management; nursing competence; tabletop exercise; site control; decontamination; disaster preparedness; impact of traditional training	Evaluation through a standardized 10‐minute tabletop exercise simulating a chemical tanker overturn with mass casualties. Performance assessed using a validated 12‐item Task‐Based Checklist (score range 0–24) with two domains: Site control (4 items) and Patient care (8 items). Inter‐rater reliability weighted kappa = 0.88–1.00.	Suboptimal performance in planning of decontamination (31.7%), debris management (32.9%), site control principles (28.6%), and decontamination skills (26.1%). High performance in establishing hazard zones (92.5%), first medical response (92.5%), and PPE use (87%). Traditional Hazmat training had no significant effect on site control (F = 0.09, *p* > 0.05) or patient care (F = 0.59, *p* > 0.05) after controlling for education, age, and gender. Higher education and male gender associated with better patient care scores; younger age associated with better site control performance.	Average
Development and Evaluation of a Multimodality Simulation Disaster Education and Training Program for Hospital Nurses	Noh et al. (2020)	Quasi‐experimental one‐group pretest–posttest design	Develop a multimodality simulation program for hospital nurses to enhance disaster competency and evaluate its effect	Hospital setting, South Korea	*N* = 40 emergency nurses	Hospital disaster nursing competencies; multimodality simulation; triage; incident command; surge capacity; life‐saving procedures; special disaster situations; crisis management; problem‐solving; technical skills; perception	12‐hour multimodality simulation training delivered Oct–Dec 2016 (4 sessions × 3 h). Modalities: virtual simulation + tabletop (triage); tabletop (incident command & surge capacity); part‐task trainers (procedures incl. PPE, ABC management, contaminated wound, splinting); full‐bodied mannequin (special situations: blast, radiation, chemical). Formative feedback/debriefing + summative assessment (Kirkpatrick).	Significant pre–post improvements across outcomes: disaster perception score increased; disaster skills (with/without PPE) increased markedly; triage performance improved in both VR and tabletop; crisis management improved (self‐assessment and observation); problem‐solving improved; high satisfaction (overall ∼9.54/10).	Average
Increasing Pediatric Emergency Nurse Readiness in Mass Casualty Incidents	Melony Murray (2025)	Quality improvement project with pre–post intervention design, one group	Assess baseline mass casualty incident readiness among pediatric emergency nurses and evaluate the impact of an educational intervention	Hospital setting, United States	Pediatric ED registered nurses (Pre, *n* = 63; Post, *n* = 64);	Pediatric disaster preparedness; nurse readiness; mass casualty incidents; simulation training	Structured educational intervention including didactic review of MCI protocols, review of supply locations, and functional simulation drills focused on zone leader roles.	Statistically significant improvement in knowledge (*p* < 0.001); 92% correctly identified supply location; 98% correctly identified zone leader role; 100% reported at least neutral preparedness; >85% achieved <10‐minute triage setup benchmark.	Average
Evaluating the Effectiveness of Psychological First Aid Training for Disaster Nursing: A Mixed‐Method Study	Bahadır Yılmaz E. (2025)	Mixed‐methods study	Evaluate the effect of PFA training on nursing students’ self‐efficacy in disaster intervention and perception of disaster preparedness	Educational setting, Turkey	*N* = 62 final‐year undergraduate nursing students (Intervention, *n* = 32; Control, *n* = 30)	Disaster preparedness; DRSES; psychosocial support; resilience; PFA perceptions (need/help/growth/struggle)	Face‐to‐face PFA training program based on WHO “Psychological first aid: Guide for field workers,” adapted/expanded (grief, PTSD symptoms, self‐care for healthcare workers); 6 sessions, 90 min each, interactive (lectures + Q&A + case presentations + group discussions using therapeutic communication).	Significant improvements in triage, incident management skills, knowledge, and confidence (*p* < 0.001), as well as in DRSES and preparedness compared with controls (*p* < 0.05), with enhanced role clarity and psychosocial response capacities.	Average
How Does Integrating ‘Disaster Nursing’ Into Nursing Curricula Impact Nursing Students' Perception of Disaster Literacy and Preparedness?	Erkin and Kiyan (2025)	Quasi‐experimental one‐group pretest–posttest design (no control group)	Investigate the effect of integrating a Disaster Nursing course on nursing students’ disaster literacy and perception of disaster preparedness	Educational setting, Turkey	*N* = 66 fourth‐year undergraduate nursing students (completed pre–post analysis, *n* = 62)	Disaster literacy; disaster preparedness perception	14‐week elective “Disaster Nursing” course (2 h/week, 2 ECTS), including lectures, case studies, group discussions, simulations/role‐play (triage), guest speakers (AFAD), projects and assignments.	Significant improvement in disaster literacy and disaster preparedness perception scores (*p* < 0.05), with gains across all sub‐dimensions and a strong positive correlation between literacy and preparedness (*r* = 0.79).	Average
Development and Evaluation of an Immersive Cinematic Escape Room for Disaster Preparedness and Self‐Efficacy Among Nurses	Hsiao et al. (2024)	Quasi‐experimental study with pretest, posttest design (intervention vs. control group comparison)	Develop and evaluate the effectiveness of an immersive cinematic escape room (ICER) approach on nurses’ disaster preparedness and self‐efficacy	Hospital setting, Northern Taiwan	*N* = 115 registered nurses (Control group: *n* = 60 Experimental group: *n* = 55)	Disaster preparedness; self‐efficacy; disaster nursing competencies; immersive learning; game‐based education	ICER disaster preparedness course (100 min) integrating cinematic movie clips, escape‐room puzzles, teamwork and simulation‐based activities covering medical station setup, ICS, personal preparedness/protection, and START mass casualty triage; compared with traditional teaching (lecture, video, discussion).	Experimental group showed significantly greater improvement in disaster preparedness over time compared to control (GEE interaction *p* < 0.05). Nurses without prior disaster training in the ICER group outperformed those with prior training. Self‐efficacy improved in both groups, with greater short‐term gains in the ICER group, though between‐group differences were not consistently statistically significant at follow‐up.	High
Active Shooter: Preparedness and Triaging Mass Casualties	Levey and Montenegro‐Montenegro (2025)	Quasi‐experimental (pre–post one‐group design)	Measure baccalaureate nursing students’ ability, willingness, and readiness to respond to an active shooting event (ASE) and to triage mass casualties	Educational setting, United States	*N* = 88 senior‐level baccalaureate nursing students	Active shooter preparedness; disaster competence; mass casualty triage; simulation‐based education	High‐fidelity active shooter simulation followed by mass casualty triage scenario, including run–hide–fight training, public safety involvement, standardized victims, moulage, and structured debriefing.	Post‐intervention scores improved across most domains. Correct responses on ASE knowledge (IS‐907) increased from 57% to 74%. Small‐to‐medium effect sizes observed for self‐regulation, ICS, triage, communication, confidence, and readiness. The intervention significantly improved students’ preparedness and triaging skills.	Average
Video‐Based Climate Change Program Boosts Eco‐Cognizance, Emotional Response and Self‐Efficacy in Rural Nursing Students: Randomised Controlled Trial	Eweida et al. (2025)	Randomized controlled trial (two‐group pretest/posttest)	To examine the effect of a Video‐Based Climate Change Program (VBCCP) on nursing students’ climate change perceptions, climate change anxiety, and environmental self‐efficacy in rural communities	Educational setting, Egypt	*N* = 140 undergraduate nursing students 2nd–4th academic year (intervention group: *n* = 70; control group: *n* = 70)	Climate change literacy/eco‐cognizance; eco‐emotional response; pro‐environmental attitudes/behaviors; environmental self‐efficacy; climate anxiety (cognitive vs functional impairment)	Intervention group: VBCCP (5 themed sessions delivered via 30–45 min videos + interactive discussions). Control group: climate change flyers. Outcomes measured at T0, T1, and T2	Compared with control, the intervention significantly improved climate change perceptions (total CCP: large effects, *d* ≈ 1.40–1.45 at T1–T2; very large gains in “causes” *d* ≈ 2.17 and “valence of consequences” *d* ≈ 1.70–1.77) and significantly increased environmental self‐efficacy (ESE: *d* ≈ 1.44 at T1; *d* ≈ 1.60 at T2). It also significantly reduced total climate anxiety (*d* ≈ 0.96 at T1; *d* ≈ 0.94 at T2) and cognitive‐impairment anxiety (*d* ≈ 1.31–1.40), while functional‐impairment anxiety did not change significantly.	High
Effect of Structured Digital‐Based Education Given to Nursing Students on Disaster Literacy And Disaster Preparedness Belief Levels	Genç et al. (2025)	Randomized controlled trial (parallel‐group pretest/posttest)	To determine the effect of structured digital‐based education on disaster literacy and disaster preparedness belief levels among nursing students	Educational setting, Turkey	*N* = 74 senior nursing students (control, *n* = 38; intervention, *n* = 36 (2 excluded from intervention for missing ≥2 consecutive weeks)	Disaster literacy, disaster preparedness beliefs, self‐efficacy (Health Belief Model)	Structured digital‐based education delivered over 8 weeks (15 sessions), combining active learning, mobile applications, computer simulation games, film analysis, online AFAD training modules, disaster drills, and a technical visit to a disaster simulation center.	Significant improvement in disaster literacy and disaster preparedness belief levels in the intervention group compared with the control group (*p* < 0.05); moderate to strong group × time effects across most outcomes; increased adoption of disaster‐related mobile applications, AFAD volunteering, and family disaster plans.	Average
Impact of Virtual Reality Training on Nurses’ Preparedness and Self‐Efficacy in Emergencies and Disasters	Alsaqer and Alhmoud (2025)	Quasi‐experimental pre–post (one‐group design)	To examine the impact of an immersive virtual reality disaster training program on emergency nurses’ preparedness and self‐efficacy	Hospital setting, Jordan	*N* = 90 registered emergency department nurses	Disaster preparedness, disaster response competencies, self‐efficacy, virtual reality simulation	Immersive VR disaster training program including theoretical instruction, VR simulation scenarios (e.g. mass casualty incidents and triage), and structured debriefing; training delivered over three sessions with immediate and two‐week follow‐up assessments.	Significant improvement in disaster preparedness (NPDCC) and disaster response self‐efficacy (DRSES) from pretest to posttest (*p* < 0.001); follow‐up scores showed slight decline but remained significantly higher than baseline (*p* < 0.001), indicating short‐term retention of skills.	Average
The Impact of Simulated Education and Training on Undergraduate Students’ Disaster Evacuation Competencies	Hawsawi et al. (2025)	Quasi‐experimental single‐group pretest and posttest	Evaluate the effectiveness of simulated education and training in enhancing undergraduate nursing students’ disaster evacuation knowledge, skills, and preparedness	Educational setting, Saudi Arabia	*N* = 119 fourth‐year undergraduate nursing students	Hospital disaster preparedness; evacuation competencies (time, process, transportation); simulation‐based education	Lecture, evacuation disaster simulation scenario (hospital fire on third floor), practical small‐group simulation (groups of 5), online pretest and posttest using the Evacuation Disaster Simulation Questionnaire (EDSQ).	Significant improvement pre–post in all subcategories: Time 1.24 ± 1.49 → 3.74 ± 1.92; Process 2.23 ± 1.05 → 3.13 ± 1.17; Transportation 3.26 ± 1.41 → 4.13 ± 1.28; overall correct answers increased 48.5% → 72.1%; highly significant improvement (*p* = 0.000) and post‐intervention total score reported as 39.66 (*p* = 0.000).	Average
The Impact of Psychological First Aid Training (RAPID‐PFA) on Self‐Efficacy, Perceived Competencies and Disaster Preparedness of Nursing Students in Tunisian Public Institutions	Mtiraoui et al. (2025)	Quasi‐experimental study with pretest, posttest design (intervention vs. control group comparison)	Evaluate the effectiveness of RAPID‐PFA training on perceived competencies, disaster preparedness, helping capacity, and self‐efficacy among nursing students	Educational setting, Turkey	*N* = 108 third‐year nursing (intervention *n* = 54; control *n* = 54)	Disaster preparedness; psychological support in disasters; perceived competence; self‐efficacy; PFA skills (RAPID model)	6‐hour immersive RAPID‐PFA workshop based on international PFA guidelines; lectures + small‐group practice + role‐play + vignette debriefing; includes “Self‐Care” module.	Baseline groups comparable (mean age ∼22; no PFA training). Intervention group improved significantly vs control in: perceived competencies (overall score ↑ from ∼64.5 to ∼118–123 across follow‐ups; *p* < 10^−^^3^; *d* ≈ 0.97), disaster preparedness (overall ↑ from ∼2.4 to ∼4.2–4.3; *p* < 10^−^^3^; *d* ≈ 0.97), helping capacity/confidence (more “very confident/confident”; *p* < 10^−^^3^), and self‐efficacy (↑ from ∼21.9 to ∼29.8–30.9; *p* < 10^−^^3^; *d* ≈ 0.86). Effects maintained at 1 and 3 months; no major drop between T2 and T3 for several outcomes.	Average
Augmented Reality in Tabletop Exercises as an Approach to Sustainable Disaster Preparedness Training: A Pilot Study	Ahayalimudin et al. (2025)	Mixed‐method study	Explore the integration of augmented reality (AR) into tabletop exercises (TTX) to enhance realism, engagement, and sustainability of disaster preparedness training	Educational setting, Malaysia	*N* = 20 undergraduate final‐year nursing students	Disaster preparedness training; tabletop exercises; augmented reality; usability; sustainability	Development of an AR‐based tabletop exercise application using Rapid Application Development (RAD); pilot testing with 20 final‐year nursing students using the System Usability Scale (SUS).	AR‐based TTX enhanced immersion, engagement, real‐time interaction, and perceived decision‐making skills compared to conventional TTX; however, usability was limited with a SUS score of 58.4 (“Not acceptable”), highlighting technical and usability challenges (device compatibility, need for technical support).	Low
Effectiveness of Skill Training for Nurse Educators in Disaster Management at Nursing Colleges	Rohini et al. (2025)	Randomized controlled trial (pretest/posttest control group)	To evaluate the effectiveness of a Hospital Disaster Management (HDM) Skill Training Program (STP) on learning (knowledge, skills, and attitudes) and behavior of nurse educators	Educational setting, India	*N* = 60 nurse educators (control, *n* = 30; intervention, *n* = 30)	HDM; disaster preparedness; nurse educators; learning and behavior	HDM Skill Training Program (STP) delivered in hybrid mode (online theory + practical simulation); 17‐unit module validated using the Delphi technique.	Significant improvements in the intervention group compared with control: Knowledge (*t* = 4.581, *p* = 0.001), skills (*t* = 3.136, *p* = 0.003), attitude (*t* = 2.093, *p* = 0.003), and behavior at 3 months (*t* = 9.34, *p* = 0.001); no loss to follow‐up.	High
The Effect of Disaster Management Training Program on Emergency Nurses’ Knowledge, Skills, and Personal Preparedness in Palestine	Issa Sa'd and Malak (2025)	Quasi‐experimentalone‐group pretest–posttest	Assess the effect of a disaster management training program on emergency nurses’ knowledge, skills, and personal preparedness for disaster management	Hospital setting, Palestine	*N* = 64 emergency registered nurses	Disaster preparedness, triage competency, personal preparedness; training effectiveness	15‐hour disaster management training over 3 days / 6 sessions: disaster management overview; START triage; decontamination and PPE for CBRNE; BLS/CPR; advanced trauma support + disaster psychology; debriefing. Teaching methods included lectures, videos, discussions, scenarios, demonstrations and re‐demonstrations (manikins).	Significant improvement pre–post in: Knowledge (pre *M* = 3.83 ± 0.82 → post *M* = 5.15 ± 0.29; *t* = −13.326, *p* < 0.001); Skills (3.70 ± 0.87 → 5.04 ± 0.27; *t* = −12.539, *p* < 0.001); Personal preparedness (3.85 ± 0.83 → 5.12 ± 0.27; *t* = −12.256, *p* < 0.001). Triage observation improved: pre 79.6% “good skills” → 100% “good skills” at 1‐month post (*Z* = −6.759; *p* < 0.001).	Average
Evaluating the Effectiveness of the Jennings Disaster Management Model on Nursing Students’ Knowledge and Self‐Efficacy	Ramadan et al. (2025)	Quasi‐experimental one‐group pre–post test	To evaluate the effectiveness of the Jennings Disaster Management Model (JDMM) on nursing students’ disaster management knowledge and self‐efficacy	Educational setting, Egypt	*N* = 648 nursing students across 4 academic levels (1st–4th years)	Disaster preparedness education; disaster management knowledge; self‐efficacy in disaster response	Structured educational intervention based on the JDMM, delivered through five interactive training sessions supported by booklets, visual aids, discussions, and constructivist learning strategies.	Significant improvement in disaster management knowledge (satisfactory knowledge increased from 0.8% to 75%, *p* < 0.001); mean self‐efficacy scores increased from 60.88 ± 15.12 to 83.27 ± 13.59 (*p* < 0.001); strong positive post‐intervention correlation between knowledge and self‐efficacy (*r* = 0.608, *p* < 0.001).	Average
Effectiveness of a Structured Disaster Management Training Program on Nurses' Disaster Readiness for Response to Emergencies and Disasters: A Randomized Controlled Trial	Lin et al. (2024)	Parallel‐group randomized controlled trial	Evaluate the effectiveness of a structured Disaster Management Training Program (DMTP) on hospital nurses’ readiness for disaster response	Medical center, Taiwan	*N* = 100 nurses (EG, *n* = 50) or control (CG, *n* = 50) group	Nurses’ disaster readiness across four domains: emergency response, clinical management, self‐protection, personal preparedness	Experimental group: regular continuing nursing education + extra 2‐day (16 h) structured DMTP delivered by transdisciplinary team using multiple strategies (lectures, simulations, problem‐solving, demonstrations, tabletop exercises, discussions, group presentations, reflections). Control: regular continuing nursing education only.	At 12 weeks, the experimental group showed a significantly greater increase in overall readiness and all four domains vs. control (GEE group×time: total readiness β = 27.3, *p* < 0.001; emergency response β = 3.7, *p* = 0.002; clinical management β = 3.7, *p* = 0.012; self‐protection β = 8.4, *p* < 0.001; personal preparedness β = 10.5, *p* = 0.003). EG total readiness mean increased 111.8→139.8 vs. CG 121.2→122.7.	High
The Impact of Surge Capacity Enhancement Training for Nursing Managers on Hospital Disaster Preparedness and Response: An Action Research Study	Shafiei et al. (2024)	Interventional pre–post action research	To assess the effect of surge capacity enhancement training for nursing managers on hospital disaster preparedness and response	Hospital setting, Iran	*N* = 20 nursing managers/supervisors/head nurses	Hospital disaster preparedness; surge capacity (staff, stuff, structures/space, system); leadership/management role; action research and continuous improvement	Two‐day workshop + round‐table exercises on risk/hazard assessment, incident command system, early warning, response plan, surge capacity; then 6‐month implementation period with biweekly disaster risk committee meetings; strategies included staffing MOUs (retired staff, students, EOC requests), stockpiling meds/equipment (≥72 h), MOUs for supplies, increased water storage, identifying/adding surge spaces (beds, school agreement, rehab dept, ED expansion), plus plans/instructions and drills.	Overall preparedness score improved from 184 (medium) pre‐intervention to 216 (high) post‐intervention (+32). Improvements across most dimensions: command and control, triage, human resources, communication, surge capacity, logistics and supply, safety and security, recovery. Minimal change in continuity of essential services. Largest gains reported for surge capacity (+10) and human resources/staff (+6).	Average
Learner Perception of Disaster Simulation Modalities: A Pilot Study	Skaltsis et al. (2024)	Comparative quasi‐experimental pilot study (post‐intervention evaluation)	To evaluate nursing students’ satisfaction, confidence, and perceived effectiveness of two disaster preparedness training modalities	Educational setting, United States	*N* = 126 senior pre‐licensure baccalaureate nursing students (58 full‐scale simulation; 57 tabletop; 11 incomplete responses)	Disaster preparedness education; learner satisfaction; self‐confidence; comparison of simulation modalities	Education; learner satisfaction; self‐confidence; comparison of simulation modalities. Two disaster preparedness training methods: (1) Full‐scale disaster simulation (3 hours, including prebrief and debrief with standardized participants); (2) Tabletop exercise (2 hours, including facilitator‐guided discussion, prebrief and structured debrief). All students received prior didactic instruction and SALT triage training.	Tabletop exercises were associated with higher perceived confidence, satisfaction, understanding of pathophysiology, and empowerment in clinical decision‐making (*p* < 0.05). Full‐scale simulation was perceived as more effective for emotional expression and constructive debriefing. Students reported higher confidence in triage skills following tabletop exercises (*p* = 0.022). Facilitator variability likely influenced outcomes.	Average
Training Public Health Nurses on Disaster Shelter Care Using a Flipped‐Classroom Approach	Holbrook et al. (2024)	Pre–post evaluation study	Evaluate the effectiveness of a flipped‐classroom disaster training program for public health nurses (PHNs), measuring changes in self‐reported knowledge and confidence	Educational setting, United States	*N* = 42 PHNs attended in‐person	Disaster shelter care; PHN disaster preparedness; flipped classroom; blended learning; knowledge + confidence/self‐efficacy; hands‐on skills + tabletop exercises	Hybrid flipped classroom: (1) ∼5‐hour online training (6 modules; quizzes with ≥80% to progress) + (2) ∼7–8‐hour in‐person training (recap + large‐group infection control tabletop + small‐group tabletop scenarios + hands‐on skill stations with standardized patients and skills lab: assessment, respiratory treatments, diabetes treatments, emergency meds incl. naloxone/epinephrine/glucagon).	Participants (*n* = 42; analyses *n* = 41) showed significant post‐training improvements: online modules increased self‐perceived knowledge across all domains (largest gains in disaster laws Δ = 1.97, *d* = 2.65; psychological first aid Δ = 1.69, *d* = 2.51), while in‐person sessions increased confidence across competencies (largest gains in naloxone Δ = 1.72, *d* = 1.50); perceived preparedness rose from 84.2% after online training to 95.1% after the hybrid program, with 78% preferring the hybrid format.	Average

*Note*: ADMR = Advanced Disaster Medical Response; AFAD = Disaster and Emergency Management Authority; ASE = active shooting event; BLS = basic life support; CBT = case‐based training; CBRNE = chemical, biological, radiological, nuclear, and explosive; CG = control group; CME = continuing medical education; CPR = cardiopulmonary resuscitation; DMTP = disaster management training program; DOH = Department of Health; DPET = Disaster Preparedness Evaluation Tool; DPP = disaster preparedness program; ED = emergency department; EDSQ = Evacuation Disaster Simulation Questionnaire; EG = experimental group; EOC = Emergency Operations Center; EPT = Emergency Preparedness Training; ESE = environmental self‐efficacy; GBT = game‐based training; GEE = generalized estimating equations; HDM = hospital disaster management; HICS = Hospital Incident Command System; ICER = Immersive Cinematic Escape Room; ICS = incident command system; IQR = interquartile range; JDMM = Jennings Disaster Management Model; MCI = mass casualty incident; MCQ = multiple‐choice question; MOUs = memoranda of understanding; MSF = Médecins Sans Frontières; NPDCC = Nurses’ Perceptions of Disaster Core Competencies; OSCE = objective structured clinical examination; PFA = psychological first aid; PHN = public health nurse; PPE = personal protective equipment; PPDTS = Psychological Preparedness for Disaster Threat Scale; PTSD = post‐traumatic stress disorder; RAD = rapid application development; RAPID‐PFA = Reflective Listening, Assessment, Prioritization, Intervention, and Disposition–Psychological First Aid; RCT = randomized controlled trial; RN = registered nurse; SALT = Sort, Assess, Life‐saving Interventions, Treatment/Transport; SD = standard deviation; SP = standardized patient; START = Simple Triage and Rapid Treatment; STP = skill training program; SUS = System Usability Scale; TTP = triage training program; TTX = tabletop exercise; VBCCP = Video‐Based Climate Change Program; VHA = Veterans Health Administration; VP = virtual patient; VR = virtual reality; WHO = World Health Organization.This table summarizes key characteristics and findings from selected studies included in the review. Only essential data are presented to ensure clarity and conciseness.

The table also includes a section dedicated to the key themes addressed in the study, the results that emerged from the analysis, and a critical assessment of the quality based on recognized criteria, allowing for the determination of the reliability and relevance of the results.

### 3.6 Data Extraction and Synthesis

The analysis was conducted by a single researcher who thoroughly reviewed and analyzed each of the selected studies. Subsequently, a narrative synthesis of the results was created. This approach allowed for the identification of common and relevant aspects among the studies, highlighting the most significant trends within the analyzed research.

## 4 Results

### 4.1 Initial Screening

The initial investigation across the three databases yielded a total of 502 articles: 289 from SCOPUS, 188 from PUBMED, and 25 from CINAHL. Four duplicates were removed, resulting in a total of 495 articles. Subsequently, a preliminary evaluation based on titles and abstracts was conducted to verify adherence to the inclusion criteria. After this phase, 454 papers were excluded. The main reasons for exclusion were the absence of quantitative data and a lack of focus on the role of nurses in disaster management.

After eliminating irrelevant papers, the full texts of the remaining 41 papers were read. Of these, five papers were excluded for the following reasons: two focused on a nonrelevant population; two were written in a language other than English; and one had an objective different from the research question. At the end of the process, 36 papers were included in the final review.

**FIGURE 1:**
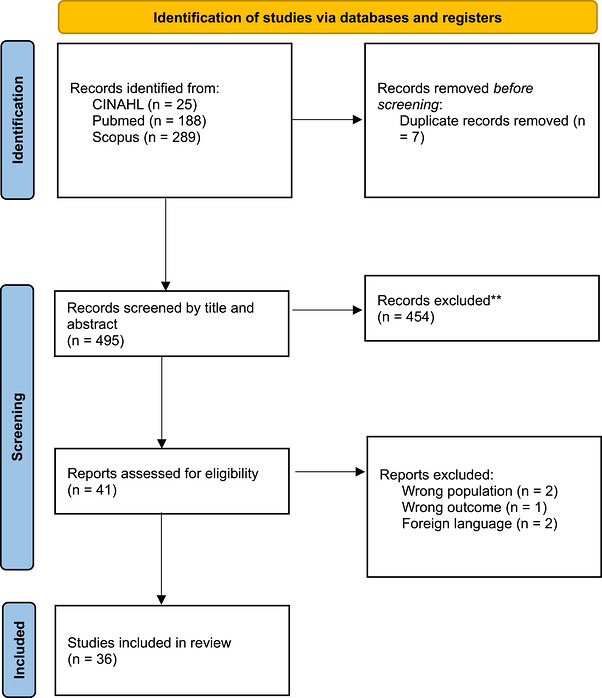
Preferred Reporting Items for Systematic Reviews and Meta‐Analysis. *Consider, if feasible to do so, reporting the number of records identified from each database or register searched (rather than the total number across all databases/registers). **If automation tools were used, indicate how many records were excluded by a human and how many were excluded by automation tools. *Source*: Page MJ, et al. BMJ 2021;372:n71. https://doi.org/10.1136/bmj.n71. This work is licensed under CC BY 4.0. To view a copy of this license, visit https://creativecommons.org/licenses/by/4.0/.

The process of identification and selection of papers was conducted in accordance with PRISMA guidelines (Page et al. 2021), ensuring transparency and methodological rigor at every stage of the review (Figure 1).

### 4.2 Characteristics of Included Studies

A total of 36 studies were included in the review (see Table 2). The overall body of evidence was predominantly interventional, evaluating the impact of an educational or training intervention using a pre–post assessment framework.

From a methodological perspective, the included studies were heterogeneous but clearly dominated by quasi‐experimental approaches. Seven studies (19.4%) were randomized controlled trials (RCTs), representing the highest level of methodological rigor within the included evidence. The largest proportion of studies consisted of single‐group quasi‐experimental pretest–posttest designs without a control group (15/36; 41.7%). These designs were particularly common in hospital‐based educational interventions and pilot implementation studies.

In addition, two studies (5.6%) employed cross‐sectional designs, providing observational data without longitudinal follow‐up. Two further studies (5.6%) adopted mixed‐methods approaches, integrating quantitative outcome measures with qualitative components to explore participants’ experiences and contextual factors related to the intervention.

Overall, the distribution of study designs indicates a field that is strongly intervention‐oriented but methodologically imbalanced. Although randomized trials accounted for nearly one‐fifth of the included studies, the substantial reliance on single‐group pre–post designs limits the strength of causal inferences. The relatively modest proportion of high‐rigor experimental studies suggests that disaster nursing education research, despite its recent expansion, continues to depend largely on designs characterized by moderate to low internal validity.

### 4.3 Temporal Distribution of the Studies

The temporal distribution of the included studies demonstrates a clear acceleration of research activity in recent years. Early contributions were sporadic, with one study published in 2006, 2013, 2015, and 2018, respectively. Research output began to increase in 2020 (*n* = 4), followed by 2021 (*n* = 3) and 2022 (*n* = 6). A marked expansion occurred in 2024 (*n* = 7), with the highest concentration of studies published in 2025 (*n* = 12), accounting for more than one‐third of the total body of evidence.

This pattern indicates a substantial post‐2020 surge in disaster preparedness education research, likely influenced by the COVID‐19 pandemic and heightened global awareness of health system vulnerability.

### 4.4 Context and Participants

Across the included studies, disaster preparedness education was delivered to a broad range of participant groups, encompassing both pre‐licensure nursing students and practicing nurses from diverse clinical and organizational settings. A total of 1228 practicing nurses were represented across the studies, alongside 1932 nursing students, highlighting a strong focus on both undergraduate education and continuing professional development.

Practicing participants included emergency nurses, public health nurses, nurse managers, and nurse educators. Specifically, one study focused on 60 nurse educators and another on 20 nurse managers, reflecting attention to leadership and educational roles within disaster preparedness.

In addition, 2276 practicing healthcare professionals from multiple disciplines were included across six studies involving mixed professional cohorts. One further study examined an interprofessional student population comprising 63 participants from different healthcare programs.

Overall, this distribution indicates that disaster preparedness education research addresses multiple stages of professional development and increasingly adopts an interprofessional approach, reflecting the collaborative nature of disaster response and emergency management.

### 4.5 Geographical Distribution

The 36 included studies were conducted across 15 countries. The United States accounted for the largest proportion of studies (*n* = 7; 19.4%), followed by Turkey (*n* = 5; 13.9%) and Iran (*n* = 4; 11.1%). Saudi Arabia and Taiwan each contributed three studies (*n* = 3; 8.3%). Jordan, Palestine, China, and Egypt were each represented by two studies (*n* = 2; 5.6%). The remaining countries—India, Malaysia, Israel, the Philippines, Brazil, and South Korea—each contributed one study (*n* = 1; 2.8%).

Two notable patterns emerge from this geographical distribution. First, disaster nursing education research appears particularly active in regions frequently exposed to natural disasters, geopolitical instability, or major public health emergencies, including the Middle East and parts of Asia. Several studies originated from countries with recent experiences of pandemics, armed conflict, or high seismic risk, suggesting that contextual vulnerability may stimulate curricular innovation and preparedness‐oriented training initiatives.

Second, European countries and Sub‐Saharan Africa are notably underrepresented, and Latin America contributed only a single study. This uneven distribution limits the global generalizability of the findings and indicates that the current evidence base may reflect region‐specific educational priorities, available resources, and national policy frameworks rather than universally implemented disaster nursing standards.

The concentration of studies within a relatively small group of countries further suggests that institutional capacity, research infrastructure, funding mechanisms, and national disaster governance models may influence both the development and dissemination of educational interventions in this field. Overall, although international interest is clearly increasing, the evidence remains geographically clustered rather than globally balanced.

### 4.6 Educational Interventions

Educational interventions demonstrated substantial heterogeneity in format, duration, technological integration, and pedagogical orientation, yet consistently aimed to strengthen disaster‐related knowledge, skills, preparedness perceptions, and self‐efficacy.

Model‐based structured programs were effective in both student and nurse populations. Training grounded in the Jennings Disaster Nursing Management Model improved preparedness perception and self‐efficacy among nursing students (Koca and Arkan 2020) and significantly enhanced knowledge and self‐efficacy in a large undergraduate cohort (Ramadan et al. 2025). Similarly, a structured Disaster Management Training Program delivered to hospital nurses resulted in significant improvements across multiple readiness domains compared with controls (Lin et al. 2024). Empowerment‐based operational exercises combining workshops, tabletop activities, and maneuvers also yielded sustained competence gains (Aliakbari et al. 2022), while surge capacity leadership training improved institutional preparedness indicators (Shafiei et al. 2024). Faculty‐focused hybrid disaster management training significantly enhanced knowledge, skills, attitudes, and behavior among nurse educators (Rohini et al. 2025).

Lecture‐based and blended approaches showed variable but generally positive effects. Traditional lecturing improved preparedness, though gains were greater when combined with tabletop exercises (Mirzaei et al. 2020). Disaster‐themed Game‐Based Training based on Precision Teaching principles demonstrated superior knowledge retention and behavioral fluency compared with case‐based methods (Masoumian Hosseini et al. 2022). Curriculum‐integrated disaster nursing courses significantly improved disaster literacy and preparedness perceptions (Erkin and Kiyan 2025). Telemedicine delivery of disaster courses proved comparable to face‐to‐face formats in knowledge acquisition (Dorigatti et al. 2018), while flipped‐classroom models combining online modules with in‐person simulation enhanced both knowledge and confidence in shelter care competencies (Holbrook et al. 2024).

Digital and online learning modalities were widely implemented. LMS‐supported online training improved preparedness perception (Koca and Arkan 2020), while structured digital education programs integrating simulations, drills, and mobile applications significantly increased disaster literacy and preparedness beliefs (Genç et al. 2025). Online educational interventions delivered via videoconferencing showed improvements in knowledge and attitudes, although some findings were not statistically significant (AlOtaibi et al. 2024; Al‐Qbelat et al. 2022). Fully online interprofessional disaster simulations enhanced collaborative competencies and satisfaction (Wong et al. 2022). Video‐based climate education broadened disaster preparedness toward eco‐cognizance, environmental self‐efficacy, and climate anxiety reduction (Eweida et al. 2025).

Simulation‐based strategies constituted the most recurrent approach. High‐fidelity disaster simulations improved triage, leadership, and readiness skills (Levey and Montenegro‐Montenegro 2025; Murray 2025), while multimodality simulation programs integrating virtual, tabletop, and procedural training significantly enhanced technical and crisis‐management competencies (Noh et al. 2020). Large‐scale competency‐based simulation curricula improved measurable performance objectives and knowledge retention (Scott et al. 2013). Virtual patient simulations demonstrated comparable effectiveness to standardized patients (Triola et al. 2006). In‐situ outbreak simulations significantly improved interdisciplinary knowledge and confidence (Babu et al. 2021). Pediatric evacuation simulations improved evacuation‐specific competencies (Hawsawi et al. 2025).

Emerging immersive technologies showed promising but mixed results. Virtual reality training improved preparedness and self‐efficacy among nurses (Zhang et al. 2021; Alsaqer and Alhmoud 2025), while immersive cinematic escape‐room approaches enhanced short‐term preparedness gains (Hsiao et al. 2024). Augmented reality–enhanced tabletop exercises increased engagement but revealed usability challenges (Ahayalimudin et al. 2025).

Tabletop exercises were also used independently to assess and improve hazardous materials response competence, highlighting specific performance gaps despite prior training (Chiang et al. 2020). Structured workshops for epidemic preparedness significantly improved knowledge and confidence (Carlos et al. 2015), and brief triage‐focused preparedness programs were associated with improved decision‐making skills (Alzahrani and Al‐Moteri 2022).

Psychological preparedness interventions represented a distinct yet complementary cluster. Face‐to‐face Psychological First Aid (PFA) programs improved disaster response self‐efficacy and psychosocial competence (Yılmaz 2025), while RAPID‐PFA training demonstrated significant improvements in perceived competencies, preparedness, helping capacity, and sustained self‐efficacy (Said et al. 2022; Mtiraoui et al. 2025). Culturally informed online interventions enhanced knowledge dimensions of cultural competence in emergencies (Kula et al. 2021).

Collectively, the evidence indicates that while no single pedagogical strategy emerged as universally superior, multimodal designs integrating didactic instruction, simulation‐based practice, technological immersion, and psychosocial training consistently produced the most comprehensive improvements across cognitive, technical, behavioral, and affective domains of disaster preparedness. The trajectory of more recent studies suggests a shift toward experiential, technology‐enhanced, and psychologically informed models of disaster nursing education.

### 4.7 Assessment Tools and Outcomes Domains

Across the 36 included studies, outcome assessment was multidimensional but highly heterogeneous. As summarized in Table 3, outcomes clustered across multiple domains, including disaster preparedness and readiness, self‐efficacy or confidence, knowledge acquisition, technical and behavioral performance, psychological preparedness, and a smaller set of organizational, ethical, and interprofessional competencies.

**TABLE 3:** Outcome domains assessed across included studies of disaster preparedness education in nursing and nursing student populations.

	Outcomes(s) measured													
Study (authors, year)	Disaster preparedness	Disaster response self‐efficacy	Interprofessional collaboration skills	Cultural competence	Psychological preparedness	Disaster attitudes and behavior	Ethical competence	Disaster knowledge	Disaster management	Critical thinking and problem‐solving	Technical skills	Confidence in safely caring	Decision‐making skills	Emergency care capability
Koca and Arkan (2020)	✓	✓												
Mirzaei et al. (2020)						✓		✓						
Masoumian Hosseini et al. (2022)						✓		✓						
Triola et al. (2006)						✓		✓			✓			
Scott et al. (2013)		✓						✓						
Carlos et al. (2015)								✓				✓		
Alzahrani and Al‐Moteri (2022)										✓			✓	
Zhang et al. (2021)	✓							✓			✓			✓
Kula et al. (2021)				✓										
Wong et al. (2022)			✓											
AlOtaibi et al. (2024)	✓					✓		✓						
Dorigatti et al. (2018)								✓						
Aliakbari et al. (2022)	✓		✓				✓		✓		✓			
Al‐Qbelat et al. (2022)	✓							✓			✓			
Said et al. (2022)					✓									
Chiang et al. (2020)											✓			
Noh et al. (2020)		✓								✓				
Babu et al. (2021)	✓					✓		✓						
Murray (2025)	✓							✓						
Yılmaz (2025)		✓			✓			✓	✓					
Erkin and Kiyan (2025)	✓													
Hsiao et al. (2024)	✓	✓												
Levey and Montenegro‐Montenegro (2025)	✓		✓			✓		✓						
Eweida et al. (2025)		✓			✓									
Genç et al. (2025)	✓													
Alsaqer and Alhmoud (2025)	✓	✓												
Hawsawi et al. (2025)	✓							✓			✓			
Mtiraoui et al. (2025)	✓	✓			✓									
Ahayalimudin et al. (2025)											✓		✓	
Rohini et al. (2025)						✓		✓			✓			
Issa Sa'd and Malak (2025)								✓	✓		✓			
Ramadan, Mohamed, and Abo‐Elmaty (2024)		✓							✓					
Lin et al. (2024)	✓													
Shafiei et al. (2024)	✓								✓					✓
Skaltsis et al. (2024)													✓	
Holbrook et al. (2024)					✓			✓			✓			

Disaster preparedness and readiness were the most frequently assessed outcomes, reported in over half of the included studies. These outcomes were commonly measured using validated multidimensional self‐report instruments (summarized in Appendix B; Supplementary Material), including the Disaster Preparedness Perception Scale for Nurses and the Disaster Response Self‐Efficacy Scale (DRSES) (Koca and Arkan 2020; Yılmaz 2025), the Disaster Preparedness Evaluation Tool (DPET) across different language versions (Al‐Qbelat et al. 2022; Issa Sa'd and Malak 2025; Zhang et al. 2021), and the Readiness for Disaster Response Scale (Lin et al. 2024). Curriculum‐based interventions additionally assessed disaster literacy and preparedness beliefs using structured literacy measures and Health Belief Model–informed tools (Erkin and Kiyan 2025; Genç et al. 2025). When reported, internal consistency was generally acceptable to excellent, although psychometric reporting was inconsistent across studies.

Knowledge‐related outcomes were also widely assessed, primarily through multiple‐choice or structured pre–post‐tests, particularly in outbreak preparedness and disaster management training (Carlos et al. 2015; Scott et al. 2013; Dorigatti et al. 2018). Most studies reported statistically significant post‐intervention gains; however, many locally developed or short instruments lacked detailed psychometric validation (Babu et al. 2021; Masoumian Hosseini et al. 2022), limiting cross‐study comparability.

Self‐efficacy and confidence outcomes were measured in a large proportion of studies, often alongside preparedness measures. These outcomes were assessed using validated tools such as the DRSES (Koca and Arkan 2020; Yılmaz 2025; Ramadan et al. 2025), General Self‐Efficacy Scales (Said et al. 2022; Hsiao et al. 2024; Mtiraoui et al. 2025), or author‐developed and single‐item measures (Carlos et al. 2015; Murray 2025). While pragmatic, brief, or modified instruments occasionally demonstrated weaker internal consistency (Levey and Montenegro‐Montenegro 2025), highlighting trade‐offs between feasibility and measurement robustness.

Objective performance‐based outcomes, including technical skills, triage accuracy, decision‐making, and emergency care capability, were less frequently assessed but provided stronger evidence of applied competence. These outcomes were evaluated through structured simulation checklists, observational tools, and OSCE‐based designs (see Appendix B in Supplementary Material) (Scott et al. 2013; Chiang et al. 2020; Noh et al. 2020; Masoumian Hosseini et al. 2022). Studies employing standardized performance metrics with reported reliability demonstrated clearer behavioral change, whereas several simulation‐based tools were locally developed and lacked formal validation (Murray 2025; Holbrook et al. 2024).

To enhance transparency, outcomes were also summarized using a semi‐quantitative approach (see Tables 2 and 3). Knowledge outcomes were assessed in 18 studies (50.0%), of which 17 reported statistically significant improvements. Disaster preparedness or readiness was also evaluated in 18 studies, with 17 showing significant post‐intervention gains and one study reporting no statistically significant change. Self‐efficacy outcomes were measured in 11 studies (30.6 %), with significant improvements observed in ten cases. Objective performance‐based outcomes were assessed in 13 studies (36.1%). These outcomes demonstrated significant improvements in 11 studies, while two reported either nonsignificant findings or equivalence between intervention modalities.

More specialized outcome domains were addressed in a smaller subset of studies. These included interprofessional collaboration skills (Wong et al. 2022), cultural competence (Kula et al. 2021), ethical competence and disaster attitudes (Aliakbari et al. 2022), and disaster management or critical thinking skills (Shafiei et al. 2024; Skaltsis et al. 2024). Psychological preparedness outcomes extended beyond confidence to include optimism, anxiety regulation, and broader mental health constructs (Said et al. 2022; Mtiraoui et al. 2025). Emerging areas further expanded preparedness to climate‐related cognition and emotional responses (Eweida et al. 2025) and technology usability in augmented reality–enhanced tabletop exercises (Ahayalimudin et al. 2025).

Although the overall pattern of findings supports the effectiveness of educational interventions, a small subset of studies reported nonsignificant, equivalent, or mixed results. Specifically, one online intervention showed no significant improvements in preparedness or attitudes (AlOtaibi et al. 2024), one randomized study demonstrated equivalence between virtual and traditional simulation modalities (Triola et al. 2006), and prior hazardous materials training was not associated with improved performance in a cross‐sectional analysis (Chiang et al. 2020). In addition, some interventions showed domain‐specific effects, such as improvements in knowledge and preparedness without corresponding gains in post‐disaster management or consistent self‐efficacy outcomes over time (Zhang et al. 2021; Hsiao et al. 2024).

Overall, while the field demonstrates increasing use of validated preparedness and self‐efficacy instruments, outcome assessment remains predominantly reliant on self‐report measures. Objective performance‐based assessments were less frequently employed but yielded stronger evidence of behavioral and skill‐based change. Substantial heterogeneity in outcome domains, measurement tools, reporting formats, and follow‐up duration precluded pooled quantitative synthesis. Future research would benefit from the adoption of standardized core outcome sets that combine validated multidimensional preparedness scales with reliability‐tested performance metrics to improve comparability and facilitate synthesis.

## 5 Discussion

The findings of this review provide a comprehensive perspective on disaster preparedness education in nursing and related healthcare populations, moving beyond effectiveness alone to identify when, for whom, and under which conditions different educational strategies are most impactful. Although the overall finding, that educational interventions improve preparedness‐related outcomes, is consistent with prior literature, a more critical synthesis reveals important nuances regarding context, learner level, duration, psychological dimensions, and organizational integration that extend beyond the simple confirmation of effectiveness. A key new insight of this review is that preparedness outcomes are optimized not by modality choice alone, but by the alignment between educational format, learner profile, contextual resources, and the integration of psychological and organizational components.

The interpretation of these findings is informed by established competency‐based frameworks in disaster nursing and healthcare emergency education. In particular, the State of the World's Nursing report (World Health Organization 2020) and the Core Competencies in Disaster Nursing framework (International Council of Nurses 2019) conceptualize preparedness as a multidimensional construct encompassing technical, ethical, psychological, leadership, and organizational competencies.

Evidence from systematic reviews and meta‐analyses has already demonstrated the overall effectiveness of simulation‐based and technology‐enhanced learning in nursing and health professions education (Cant and Cooper 2017; Ilgen et al. 2013). Building on this consolidated evidence, the present review extends prior work by clarifying how educational effectiveness varies according to learner level, contextual resources, and targeted outcome domains, rather than modality alone.

Effectiveness appears to be context‐ and learner‐dependent, with no single educational modality consistently superior, and outcomes shaped by the alignment between training format, learner characteristics, and implementation context. As shown in the semi‐quantitative synthesis and across included studies, several interventions reported equivalent or nonsignificant effects depending on the educational format (AlOtaibi et al. 2024; Triola et al. 2006; Chiang et al. 2020). For instance, telemedicine‐ and online‐based approaches generally demonstrated outcomes comparable to face‐to‐face training across selected domains rather than clear superiority (Dorigatti et al. 2018; Wong et al. 2022), while some online interventions produced limited or nonsignificant improvements (AlOtaibi et al. 2024). Conversely, simulation‐based and multimodal interventions more consistently improved applied competence and performance‐related outcomes (Scott et al. 2013; Noh et al. 2020; Masoumian Hosseini et al. 2022; Murray 2025).

Furthermore, emerging leadership models emphasize preparedness as an adaptive, system‐level capability grounded in evidence‐based practice and crisis leadership (Stetler et al. 2014). These findings resonate with adaptive leadership models in healthcare emergencies, which conceptualize preparedness not as a fixed set of competencies but as the capacity of individuals and systems to adjust roles, decision‐making processes, and coordination mechanisms in response to evolving uncertainty. Framed within these perspectives, our findings highlight that disaster preparedness education is most effective when aligned with organizational structures and leadership processes that support resilience, coordination, and adaptive decision‐making under uncertainty.

First, effectiveness appears to be context‐sensitive rather than format‐dependent. Simulation‐based interventions consistently improved technical skills, triage performance, and crisis‐management behaviors (Scott et al. 2013; Noh et al. 2020; Levey and Montenegro‐Montenegro 2025; Murray 2025), yet their impact was particularly pronounced in hospital‐based samples and among practicing nurses exposed to real clinical uncertainty (Lin et al. 2024; Aliakbari et al. 2022). In these contexts, structured training programs that combined operational drills, leadership roles, and incident command rehearsal produced sustained improvements across competence domains. By contrast, in undergraduate populations, structured curricular integration and model‐based frameworks—such as programs grounded in the Jennings Disaster Nursing Model—were highly effective even when delivered through blended or online modalities (Koca and Arkan 2020; Ramadan et al. 2025; Erkin and Kiyan 2025; Genç et al. 2025). This suggests that learner level moderates optimal format: students benefit from scaffolded conceptual integration, whereas practicing professionals may require experiential reinforcement targeting applied decision‐making and system coordination.

Second, duration and pedagogical structure appear to influence outcome stability. Short, intensive workshops improved knowledge and confidence (Carlos et al. 2015; Alzahrani and Al‐Moteri 2022), but more sustained gains were observed in multisession or reinforced programs with follow‐up components (Aliakbari et al. 2022; Mtiraoui et al. 2025). Game‐based Precision Teaching demonstrated superior retention compared to case‐based approaches (Masoumian Hosseini et al. 2022), highlighting the importance of repeated performance rehearsal. Similarly, multimodal curricula integrating simulation, structured debriefing, and competency‐based feedback (Scott et al. 2013; Noh et al. 2020) were more likely to demonstrate measurable behavioral change rather than improvements limited to self‐perception. These findings suggest that iterative experiential exposure and structured feedback loops are critical mechanisms underlying durable preparedness.

Third, while digital and immersive technologies were widely adopted, their added value appears contingent on feasibility and infrastructure. Virtual reality training improved preparedness and self‐efficacy (Zhang et al. 2021; Alsaqer and Alhmoud 2025), and immersive or cinematic escape‐room approaches enhanced engagement and short‐term gains (Hsiao et al. 2024). However, augmented reality tabletop applications revealed usability barriers (Ahayalimudin et al. 2025), underscoring that technological sophistication does not automatically translate into superior outcomes. Telemedicine and online simulation demonstrated comparable knowledge gains to in‐person formats (Dorigatti et al. 2018; Wong et al. 2022), suggesting that in low‐resource or geographically dispersed settings, scalable digital solutions may offer pragmatic equivalence without requiring high‐cost infrastructure. Thus, format superiority may be less relevant than alignment between resources, learner needs, and contextual constraints.

A central conceptual insight emerging from this review concerns the underrecognized psychological and organizational dimensions of preparedness. Beyond technical readiness, several studies highlight preparedness as a multidimensional construct encompassing emotional resilience, ethical responsibility, and system‐level engagement. This broader framing is consistent with recent evidence emphasizing that disaster education should integrate psychosocial, cultural, and environmental considerations alongside acute response competencies (Mani et al. 2025). Such findings reinforce the importance of conceptualizing preparedness not only as individual skill acquisition but as an adaptive capacity shaped by cognitive, behavioral, and organizational factors.

PFA and RAPID‐PFA interventions produced significant improvements in self‐efficacy, optimism, anxiety regulation, and perceived competence (Said et al. 2022; Yılmaz 2025; Mtiraoui et al. 2025). These findings indicate that disaster readiness is not solely technical but deeply behavioral and affective. Cultural competence training further expanded preparedness into ethical and contextual awareness (Kula et al. 2021), while climate‐related disaster education addressed environmental self‐efficacy and anxiety (Eweida et al. 2025). Collectively, these studies suggest that preparedness encompasses cognitive mastery, emotional resilience, and adaptive coping under uncertainty.

At the organizational level, interventions that embedded training within institutional governance structures demonstrated system‐level gains. Leadership‐focused surge capacity planning improved hospital preparedness indices (Shafiei et al. 2024), and faculty‐targeted disaster management programs enhanced educator capacity and behavioral outcomes (Rohini et al. 2025). Such findings align with theories of institutional learning: preparedness strengthens when simulation and training are integrated into organizational processes rather than confined to individual skill acquisition. From a systems perspective, disaster preparedness can therefore be conceptualized as a layered construct operating at individual, team, and institutional levels. At the individual level, psychological resilience and adaptive decision‐making under stress are critical. At the team level, leadership, communication, and coordinated role clarity determine functional response capacity. At the institutional level, simulation, debriefing, and governance integration serve as mechanisms of organizational learning that transform episodic training into sustained system resilience.

Importantly, the review also highlights methodological limitations. These findings are consistent with prior Saudi literature identifying structural gaps in disaster nursing education. A scoping review of disaster nursing education in Saudi Arabia reported insufficient simulation‐based training, limited formal curricular integration, and difficulty accessing up‐to‐date educational resources (Brinjee et al. 2021). Similarly, empirical evidence from emergency nurses in Taif identified incident management systems, disaster triage, and disaster drills as the most pressing training needs, particularly among less experienced nurses (Brinjee et al. 2021). Together, these findings suggest that while intervention effectiveness is frequently demonstrated in controlled studies, systemic educational infrastructure and competency standardization remain unevenly implemented.

Although nearly half of the studies incorporated comparison groups, many relied on self‐report measures of preparedness and confidence. Objective performance‐based assessments, such as structured checklists and OSCEs, provided stronger evidence of applied competence (Scott et al. 2013; Chiang et al. 2020; Masoumian Hosseini et al. 2022), yet standardized core outcome sets were lacking. This heterogeneity limits cross‐study comparability and complicates the synthesis of effect magnitude. Taken together, these findings support a shift from intervention‐centric to system‐aligned models of disaster preparedness education.

Overall, the novel contribution of this review lies in identifying that no single educational strategy is universally superior; rather, effectiveness emerges from alignment between format, learner profile, contextual resources, and integration of psychological and organizational components. The most comprehensive improvements were observed in multimodal programs combining didactic instruction, experiential simulation, reflective debriefing, and psychosocial training (Lin et al. 2024; Noh et al. 2020; Mtiraoui et al. 2025). Recent studies (2024–2025) reflect a shift toward immersive, resilience‐oriented, and systems‐integrated models (Levey and Montenegro‐Montenegro 2025; Hsiao et al. 2024; Murray 2025; Eweida et al. 2025), suggesting an evolution in disaster nursing education from knowledge transmission to adaptive capacity development.

In conclusion, disaster preparedness education in nursing is most effective when it transcends technical skill acquisition and incorporates behavioral resilience, leadership under stress, interprofessional coordination, and institutional embedding. Future research should prioritize longitudinal designs, standardized performance metrics, and explicit integration of psychological and organizational preparedness constructs to strengthen both internal validity and practical impact.

### 5.1 Limitations

This REA has several inherent limitations related to the search strategy, methodological heterogeneity, and representativeness of the available evidence. The literature search was restricted to three databases (Scopus, PubMed, and CINAHL), which may have led to the exclusion of relevant studies indexed in other sources, thereby introducing potential selection bias.

Furthermore, the methodological quality of the included studies was heterogeneous, with a substantial proportion classified as moderate quality. This variability limits the robustness of causal interpretations and suggests that conclusions regarding the effectiveness of specific educational interventions should be interpreted with caution. In particular, the predominance of quasi‐experimental and single‐group pre–post designs increases the risk of overestimating intervention effects due to self‐report bias and limited control of confounding variables.

Although studies were drawn from different countries, their geographical distribution was uneven, with a predominance of high‐ and middle‐income settings. This limits the global generalizability of the findings, as disaster preparedness priorities, educational infrastructures, and resource availability vary considerably across contexts. Consequently, the conclusion that multimodal and simulation‐based strategies are broadly effective may not fully apply to low‐resource or underrepresented regions, where infrastructure, faculty expertise, and technological access differ substantially.

In addition, the exclusion of two studies published in Arabic and Chinese, due to the authors’ inability to conduct a rigorous critical appraisal, may have further reduced the cultural and contextual diversity of the evidence base. This language restriction may have led to underrepresentation of locally developed educational models or context‐specific preparedness strategies, potentially reinforcing an evidence base skewed toward English‐language and internationally indexed research.

Taken together, these limitations suggest that while the review identifies consistent patterns of educational effectiveness, the conclusions should be interpreted as indicative rather than universally generalizable. Future syntheses would benefit from broader database coverage, multilingual collaboration, and stronger inclusion of studies from low‐ and middle‐income settings to enhance global representativeness and conceptual robustness.

### 5.2 Implications for Nursing and Health Policy

The findings of this rapid evidence assessment have important implications for nursing practice, nursing education and health policy. Disaster preparedness should be recognised as a core component of nursing competence, rather than an optional or specialist area of training. Nurses are central to preparedness, response and recovery during disasters, and therefore require ongoing opportunities to develop the knowledge, technical skills, confidence, leadership, communication and psychological resilience needed to respond effectively in complex and rapidly changing situations. For nursing practice, the findings suggest that disaster preparedness training should be embedded within clinical services and linked to local emergency planning. Training should not be limited to theoretical knowledge but should include realistic, simulation‐based and scenario‐based learning that enables nurses to practise decision‐making, triage, communication, interprofessional coordination and safe care delivery under pressure. Regular refresher training, debriefing and reflective learning should also be incorporated to support confidence, emotional resilience and retention of competencies over time. For nursing education, disaster preparedness should be integrated into undergraduate nursing curricula, postgraduate education and continuing professional development. Programmes should be competency‐based, aligned with measurable learning outcomes and adapted to the learner’s level, clinical role and local context. Interprofessional education should be included, as disaster response requires coordinated working across nursing, medicine, public health, emergency services, management and community partners. Simulation, virtual reality, tabletop exercises and technology‐enhanced learning may be particularly useful when combined with structured debriefing and assessment. For health policy, the findings highlight the need for national and organisational frameworks that mandate and support disaster preparedness education for nurses and nursing students. Policy makers, regulators, education providers and healthcare organisations should work together to establish minimum standards for disaster nursing education, including core competencies, assessment approaches and requirements for continuing professional development. Such frameworks should also consider cultural competence, psychological preparedness, staff wellbeing, resource availability and the needs of low‐resource settings. Future policy and research should also prioritise the use of validated assessment tools, objective performance‐based measures and longitudinal follow‐up to determine whether educational gains are sustained and transferred into practice. Evaluating cost‐effectiveness and scalability will be important to support sustainable implementation across different healthcare systems. Overall, strengthening disaster preparedness education is essential for developing a nursing workforce that is technically competent, emotionally resilient, culturally responsive and able to contribute effectively to health system resilience during crises.

## 6 Conclusions

Advanced educational approaches, particularly simulation‐based, technology‐enhanced, and multimodal programs, not only strengthen technical preparedness but also positively shape nurses’ attitudes, confidence, and readiness to face emergencies. Integrating these training models into undergraduate curricula and continuing professional education is essential to ensure comprehensive and sustainable preparedness in an increasingly vulnerable and disaster‐prone global context.

However, effective disaster education extends beyond technical skill acquisition. Culturally sensitive competencies are fundamental to delivering inclusive, context‐responsive care during crises. The challenge lies in achieving a dynamic balance between theory and practice—combining innovative methodologies with established pedagogical strategies to maximize both knowledge retention and applied performance.

The evidence demonstrates that educational programs generally improve knowledge, preparedness perceptions, and self‐efficacy, with growing support for measurable performance gains when objective assessments are incorporated. Crucially, effectiveness is not determined by modality alone. It depends on alignment between teaching strategy, learner level, training duration, and available resources, as well as on the explicit integration of psychological resilience and organizational learning principles.

Ultimately, strengthening disaster preparedness requires more than better educational techniques. It demands institutional commitment to embedding training within systems that reinforce competence over time—transforming episodic learning into sustained adaptive capacity. Only through this integrated, system‐level approach can nursing education cultivate a workforce that is not only technically prepared but also resilient, responsive, and capable of leading in times of crisis.

## Author Contributions

Conceptualization: AB, GC, RW, LS, MZ, LD, and SM. Study design: AB, GC, RW, LS, MZ, and LD. Data collection: LD, SM, CC, and MDN. Data analysis: LD, SM, CC, RW, MDN, and GC. Study supervision: AB, GC, RW, LS, and MZ. Manuscript writing: LD, SM, and GA. Critical revisions for important intellectual content: AB, GC, RW, LS, MZ, and GA.

## Funding

The authors have nothing to report.

## Ethics Statement

Ethical approval was not required for this study, as it involved an REA of previously published research and did not involve human participants.

## Conflicts of Interest

The authors declare no conflicts of interest.

## Supporting information




**Supporting File 1**: inr70193‐AppendixA.docx

## Data Availability

Data sharing not applicable to this article as no datasets were generated or analyzed during the current study.
